# Supplementary material for: The Rqc2/Tae2 subunit of the ribosome-associated quality control (RQC) complex marks ribosome-stalled nascent polypeptide chains for aggregation
Source: eLife. 2016 Mar 4;5:e11794. doi: 10.7554/eLife.11794 (PMC4805532; doi:10.7554/eLife.11794)
Supplement: Supplementary file 1. — K0 and K12 reporters were Flag IP’ed from WCE and analyzed by mass spectrometry. Peptide counts of co-IP’ed chaperones are shown. As represented in diagrams in Figure 1A, the K0 and K12 reporters consist of a GFP-Flag-HIS3 backbone. Thus, HIS3 peptide counts, which were the most abundant in the analyses, are presumed to be derived from the IP’ed reporter. We note that these analyses have limited ability to distinguish among homologous proteins with high degree of similarity, such as Ssa1/Ssa2, Hsc82/Hsp82, and Ssb1/Ssb2. DOI: http://dx.doi.org/10.7554/eLife.11794.012 [file elife-11794-supp1.doc]

**Supplementary file 1 (Table SI).**

| **Gene** | **Accession** | **ltn1Δ K0** | **ltn1Δ K12** | **Description** |
| --- | --- | --- | --- | --- |
| **HIS3 (reporter)** | **YOR202W** | **1578** | **924** | "Imidazoleglycerol-phosphate dehydratase, catalyzes the sixth step in histidine biosynthesis; mutations cause histidine auxotrophy and sensitivity to Cu, Co, and Ni salts; transcription is regulated by general amino acid control via Gcn4p" |
| SSA1 | YAL005C | 14 | 64 | "ATPase involved in protein folding and nuclear localization signal (NLS)-directed nuclear transport; member of heat shock protein 70 (HSP70) family; forms a chaperone complex with Ydj1p; localized to the nucleus, cytoplasm, and cell wall" |
| SSA2 | YLL024C | 14 | 62 | "ATP binding protein involved in protein folding and vacuolar import of proteins; member of heat shock protein 70 (HSP70) family; associated with the chaperonin-containing T-complex; present in the cytoplasm, vacuolar membrane and cell wall" |
| **SIS1** | **YNL007C** | **0** | **52** | "Type II HSP40 co-chaperone that interacts with the HSP70 protein Ssa1p; not functionally redundant with Ydj1p due to due to substrate specificity; shares similarity with bacterial DnaJ proteins" |
| SSB2 | YNL209W | 2 | 36 | "Cytoplasmic ATPase that is a ribosome-associated molecular chaperone, functions with J-protein partner Zuo1p; may be involved in the folding of newly-synthesized polypeptide chains; member of the HSP70 family; homolog of SSB1" |
| SSB1 | YDL229W | 2 | 33 | "Cytoplasmic ATPase that is a ribosome-associated molecular chaperone, functions with J-protein partner Zuo1p; may be involved in folding of newly-made polypeptide chains; member of the HSP70 family; interacts with phosphatase subunit Reg1p" |
| HSC82 | YMR186W | 4 | 30 | "Cytoplasmic chaperone of the Hsp90 family, redundant in function and nearly identical with Hsp82p, and together they are essential; expressed constitutively at 10-fold higher basal levels than HSP82 and induced 2-3 fold by heat shock" |
| HSP82 | YPL240C | 4 | 27 | "Hsp90 chaperone required for pheromone signaling and negative regulation of Hsf1p; docks with Tom70p for mitochondrial preprotein delivery; promotes telomerase DNA binding and nucleotide addition; interacts with Cns1p, Cpr6p, Cpr7p, Sti1p" |
| SSA4 | YER103W | 0 | 12 | "Heat shock protein that is highly induced upon stress; plays a role in SRP-dependent cotranslational protein-membrane targeting and translocation; member of the HSP70 family; cytoplasmic protein that concentrates in nuclei upon starvation" |
| YDJ1 | YNL064C | 0 | 9 | "Protein chaperone involved in regulation of the HSP90 and HSP70 functions; involved in protein translocation across membranes; member of the DnaJ family" |
| SSA3 | YBL075C | 0 | 9 | "ATPase involved in protein folding and the response to stress; plays a role in SRP-dependent cotranslational protein-membrane targeting and translocation; member of the heat shock protein 70 (HSP70) family; localized to the cytoplasm" |
| AHA1 | YDR214W | 0 | 4 | "Co-chaperone that binds to Hsp82p and activates its ATPase activity; similar to Hch1p; expression is regulated by stresses such as heat shock" |
| HSP42 | YDR171W | 0 | 2 | "Small heat shock protein (sHSP) with chaperone activity; forms barrel-shaped oligomers that suppress unfolded protein aggregation; involved in cytoskeleton reorganization after heat shock" |
