# Supplementary material for: The Rqc2/Tae2 subunit of the ribosome-associated quality control (RQC) complex marks ribosome-stalled nascent polypeptide chains for aggregation
Source: eLife. 2016 Mar 4;5:e11794. doi: 10.7554/eLife.11794 (PMC4805532; doi:10.7554/eLife.11794)
Supplement: Supplementary file 2. — DOI: http://dx.doi.org/10.7554/eLife.11794.013 [file elife-11794-supp2.doc]

**Supplementary file 2 (Table SI). Strain genotypes.**

| Strain | Genotype | Source |
| --- | --- | --- |
| BY4741 *ltn1*Δ | MATa: *his3*Δ1; *leu2*Δ0; *met15*Δ0; *ura3*Δ0; *ltn1*Δ::KanMX6 | Thermo Scientific |
| BY4741 *hel2*Δ | MATa: *his3*Δ1; *leu2*Δ0; *met15*Δ0; *ura3*Δ0; *hel2*Δ::KanMX6 | Thermo Scientific |
| BY4741 *rqc2*Δ | MATa: *his3*Δ1; *leu2*Δ0; *met15*Δ0; *ura3*Δ0; *rqc2*Δ::KanMX6 | Thermo Scientific |
| BY4741 *rqc1*Δ | MATa: *his3*Δ1; *leu2*Δ0; *met15*Δ0; *ura3*Δ0; *rqc1*Δ::KanMX6 | Thermo Scientific |
| BY4741 *ltn1*Δ*hel2*Δ | MATa: *his3*Δ1; *leu2*Δ0; *met15*Δ0; *ura3*Δ0; *hel2*Δ::KanMX6; *ltn1*Δ::His3MX6 | This study |
| BY4741 *ltn1*Δ*rqc2*Δ | MATa: *his3*Δ1; *leu2*Δ0; *met15*Δ0; *ura3*Δ0; *rqc2*Δ::KanMX6; *ltn1*Δ::His3MX6 | This study |
| BY4742 Ltn1ΔRING-HA | MATα: *his3*Δ1; *leu2*Δ0; *lys2*Δ0; *ura3*Δ0; *ltn1*Δ 4500-3xHA::KanMX6 | Bengtson and Joazeiro, 2010 |
| DS10 *ltn1*Δ | MATa: *trp1*Δ*; lys1; lys2; ura3-52; leu2-3,112; his3-11,15; ltn1*Δ::His3MX6 | This study |
| W303 Tet-Sis1 | MATa; *leu2-3,112*; *trp1-1*; *can1-100*; *ura3-1*; *ade2-1*; *his3-11,15*; *sis1*Δ::*LEU2*; pCM184 tetO7-*SIS1* (*TRP1*) | Aron et al., 2007 |
| W303 Tet-Sis1 *ltn1*Δ  | MATa; *leu2-3,112*; *trp1-1*; *can1-100*; *ura3-1*; *ade2-1*; *his3-11,15*; *sis1*Δ::*LEU2*; pCM184 tetO7-*SIS1* (*TRP1*) *ltn1*Δ::KanMX6 | This study |
